# Dementia and the prison population: identifying need

**DOI:** 10.12968/bjnn.2023.19.5.178

**Published:** 2023-10-02

**Authors:** V Blundell-White, J Harrison, J Hill

**Affiliations:** 1 Lancashire and South Cumbria NHS Foundation Trust; 2 Synthesis, Economic Evaluation and Decision Science Group, University of Central Lancashire

## Abstract

Older adults are the fastest growing group within the UK prison population yet there continues to be a lack of national strategy to ensure consistent care is provided to this group, in line with the general population. The number of older adults with dementia is also rising globally however the prevalence of dementia in prisons remains largely unknown. The impact of dementia on older adults in prison is also largely unexplored. Subsequently a review by Brooke et al. 2020 was undertaken to identify prevalence of dementia in the UK prison population and to explore how such prisoners are assessed, diagnosed, treated and supported. This article critically appraises and evaluates the systematic review by Brooke et al. 2020, and expands upon the findings in context to practice.

## Introduction

The number of people living with dementia globally is rising (GBD 2022) with an estimated 900,000 older people living with the condition in the UK alone (Wittenberg et al. 2019). A person with dementia may experience memory loss, problems with communication, impaired reasoning and difficulties with daily living skills (Azam et al. 2016). This can result in behaviour changes, the ability to live independently and may affect social relationships (RCN 2021). In the prison population, one study found that 8% of the older prison population had suspected dementia or mild cognitive impairment (Forsyth et al. 2020). There remains however a lack of information on prevalence within the prison population (Brooke et al. 2020).

It is acknowledged that prisoners have an accelerated aging process, with health-related needs advanced by around 10 years, compared to the general population (RCN 2021). This could be explained by poor life-style choices and social deprivation, which commence prior to and during a prison sentence (HoC 2020). Older prisoners commonly suffer poor mental and physical health and have specific health and social care needs associated with older age that are exacerbated by the experience of being imprisoned (CQC 2019). The additional demands placed on prison health and social care is one of the major challenges created by an ageing prison population (HoC 2020). In a 2013 inquiry of the impact of an ageing prison population, Age UK (2013) expressed concern that dementia diagnoses remain largely overlooked in prisons compared to the general population, and it has been argued that the structured and predictable format of prison can mask signs of deterioration and obstruct diagnosis (CQC 2019, Moll 2013). Given that the number of prisoners aged 60 or above has increased by 82% in the previous decade and older prisoners are the fastest growing group in the prison population (Age UK 2013, HoC 2020), this will remain an ongoing concern.

Within prison, access to memory services and assessment is limited and may be hindered by security measures required for an individual to attend and differences in assessment setting from the prison environment, causing long delays (CQC 2019). Prison staff can also lack awareness of the condition (HoC 2020). There is therefore a need to further understand the impact of dementia in a custodial setting to fully support people with dementia in prison and for prison staff to recognise symptoms of dementia and effectively manage this condition. A systematic review by Brooke et al. (2020) aimed to identify the prevalence of dementia in the prison setting, and how prison, health and social care providers assess, diagnose, treat, support and care for prisoners with dementia. This commentary aims to critically appraise the methods used within the review by Brooke et al. 2020 and reflect on the applicability of these findings to practice.

## Methods of the review by Brooke et al. 2020

A multi-database search was completed from inception up to December 2017 and supplemented by searches of reference lists, grey literature and relevant journals. Searches were restricted to the English language. Studies were included in the systematic review if they presented primary data relating to prisoners with cognitive impairment or dementia, and within a prison setting. Studies were excluded if they did not identify dementia or did not include primary outcome data. Two reviewers independently screened studies and data extraction was completed by one reviewer and checked by another. A number of tools were used for quality assessment due to the different methodological approaches identified. No information was given regarding the number of reviewers who undertook the quality assessment of included studies. Key issues and themes were extracted from the data and thematically synthesised using an approach of coding, development of themes and analytical interpretation. This process was completed by one reviewer and discussed with two others. Due to the variation in approaches of included studies, a meta-analysis was not possible.

## Results

A total of 2469 articles were identified of which 68 were assessed for full text eligibility and 10 included in the qualitative synthesis. Included studies were conducted in: USA (n=3), UK (n=3), Australia (n=2), France (n=1) and Sweden (n=1) and explored different aspects of dementia in the prison setting. These included prevalence, knowledge of prison personnel (correctional officers and legal professionals) and identification of needs for prisoners with cognitive impairment or dementia. Overall, 1056 prisoners were included in the review of whom the majority were male aged 50 or over. Healthcare professionals (n=83), correctional officers (n=71) and legal staff (n=72) were also represented. Included study designs were semi-structured interviews, validated questionnaires, case note evaluations, mixed methods, round table and Delphi design. Assessment of study quality found only minor elements were identified.

Three main themes identified from this review were; prevalence of dementia in prison populations; identification of older prisoners’ needs; knowledge of correctional officers and legal professionals. A summary of the main findings for each theme can be found in Table 1.

### Prevalence

The prevalence of dementia in Prisons ranged from 0.8% to 18.8%. The different rate between studies appeared to be due to different prison populations in each prison and the use of different screening and diagnostic tools. Some studies in the review used standardised tools for identifying psychiatric and personality disorders whereas others applied specific screening tools to identify cognitive impairment. Different interpretations of scoring on the MMSE may also have led to a disparity between studies.

### Identification of older prisoners needs

Identification of older prisoners needs along with how to improve the quality of care for prisoners with dementia was explored by experts in prison healthcare and healthcare professionals. Studies in the review identified the need for functional requirements; to support older prisoners living independently and a framework for staff to manage and care for older prisoners with dementia. Specific needs to this population included: development of appropriate accommodation, an MDT approach, and specialist care and environments. Healthcare professionals also acknowledged a need for identifying prisoners with cognitive impairment and to help achieve this, development of suitable cognitive screening tools. A need for a framework to support cognitive screening and referral practices in a transparent and consistent manner was also recognised.

### Knowledge of correctional officers and legal professionals

Within the review, a comparison of the identification of prisoners with disabilities by correctional officers to information from prisoner case note reports found that officers reported higher rates of disability than acknowledged in the case notes. Prisoners identified as having a disability by officers tended to be older, have been hospitalised within the last 2 years and have a hearing impairment. There were no significant differences found between the number of prisoners diagnosed with dementia who were either known or unknown to their assigned officer. Amongst legal professionals, there was a lack of knowledge on how to identify and respond to cognitive impairment and only 29% had received any age-related training. Included studies also identified a need for robust training programmes to include staff knowledge and identification of the aging process, cognitive impairment and disabilities relating to aging. The use of a comprehensive, standardised checklist was recommended by studies to support these needs.

### Commentary

Using the Joanna Briggs Critical Appraisal Tool for Systematic reviews (JBI 2017), 8 of the 11 criteria were found to be satisfactory for this review. The criteria that were unmet or unclear related to: extraction of study data (completed independently by one author only and no piloting reported), critical appraisal (unclear how many authors undertook this task), and specific directives for new research (the authors discuss where evidence is lacking within the discussion, however there are no clear recommendations for future research). In addition, despite the indication that a quality assessment of individual studies was undertaken, it is unclear of the outcome, stating that only minor elements were identified. It is also worth noting that the themes identified in the data synthesis process would have benefitted from greater transparency as to how they were developed, by for example providing supportive quotes. Overall, the systematic review provides a comprehensive summary of the available data that address the question of interest. However, some caution should be applied when interpreting the results, due to the limitations reported. Further evidence will therefore be discussed below when considering the review’s findings in view of recommendations for practice.

The three themes identified within the review suggest three main areas for improvement within prison systems: screening and referral processes for prisoners with dementia, improvement of prison environment for prisoners with dementia, and appropriate staff training. On screening and referral processes for dementia, the Royal College of Nursing (RCN) Dementia Care Principles for People in Prison state that the prison population should have parity of service with the community including screening, prompt identification and accurate, timely diagnosis (RCN 2021). An assessment for dementia could therefore be considered as part of routine consultation during a prison stay, taking place in a conducive environment for communication, and ensuring the person is able to see and hear (RCN 2021). In terms of a specific screening tool, it is advised that clinical staff working with the prison population should be familiar with dementia screening tools, using agreed approaches and processes for screening and assessment (RCN 2021). NICE guidelines for dementia (NICE 2018) also suggest that in non-specialist settings a validated, brief cognitive assessment should be utilised such as the 10-point cognitive screener (10-CS), the 6-item cognitive impairment test (6CIT), the 6-item screener, the Memory Impairment Screen (MIS), and the Mini-Cog and Test your Memory (TIM). Specific to an older prison population, a study identified that the Montreal Cognitive Assessment (MOCA) screening test was found to be more effective than (6CIT) for identifying mild cognitive impairment or dementia (Forsyth et al. 2020). However, it should be considered that the MOCA also requires training for staff prior to its use to improve validity and reliability of the test (Nasreddine 2019). Greater consensus on choice of screening tool for the prison population would also help to identify accurate prevalence rates which varied widely in the review.

The review also identified a need for improving the prison environment for those with dementia or cognitive impairment. Following an inquiry of prisons in England and Wales, the justice committee recommended provision of reasonable adjustments and adaptations to meet the needs of older prisoners (HoC 2020). This includes specifically adapted accommodation, yet existing prison estate is often unsuited to the needs of older prisoners and only half of prisons surveyed had made modifications to the physical environment specifically for prisoners with dementia or mild cognitive impairment (Forsyth et al. 2020). It is therefore recommended that new prisons develop additional accommodation specifically adapted for older prisoners (HoC 2020). In addition, the RCN Dementia Care Principles for People in Prison state that unfamiliar environments can be difficult for people with dementia or cognitive impairment and that prison environments can be confusing, noisy and difficult to navigate (RCN 2021). They recommend that environments should be dementia friendly, support independence and wellbeing and take into account sensory and mobility needs including a person’s ability to navigate. This can be supported by reducing moves between environments, and dementia friendly design features including appropriate signage, lighting and noise minimisation (RCN 2021). These dementia supportive elements could be included within designs for new prisons and adapted to older prisons, following an individual risk assessment.

Finally, the review identified a need for prison and legal personnel to receive training on dementia. Other studies have also identified issues related to the training of prison personnel such as limited knowledge and awareness of dementia, and lack of resources and training available to prison staff (Chamberlain & Harrison-Dening, 2020; Forsyth et al. 2020; Surr & Gates 2017). There is a need for all personnel within the prison environment to receive education, training, policies and guidelines to support prisoners with dementia (Brooke & Jackson 2019, Forsyth et al. 2020, RCN 2021). It is also recommended that prison healthcare teams have a designated older prisoner lead and that training on age-related health issues is available to all staff (HoC 2020). There are no training standards currently used in UK prisons, but a framework such as the Dementia Training Standards Framework (Skills for Health & HEE, 2018) details essential skills and knowledge for the health and social care workforce and may be adaptable for dementia training in prisons. Dementia education workshops in the prison environment have been well received by staff and prisoners (Brooke & Rybacka 2020), as have dementia information sessions, increasing participant’s knowledge, confidence and understanding of dementia (Treacy et al. 2019). Further suggested content for training programmes could include issues for rapid and/or further referral and ways of managing dementia associated behaviours (Du Toit et al. 2019). Once a training programme for staff is identified, it should be delivered to all interagency and multidisciplinary groups at regular intervals to encourage shared understanding and ownership of the issue (Forsyth et al. 2020).

An inquiry by the Justice Committee in both 2013 and 2020, recommended that the Ministry of Justice produce a national strategy for older prisoners to provide minimum standards, leading to effective and equitable care (HoC 2020). There is currently no national strategy for older prisoners, however the HoC report continues to recommend a strategy that includes the following: provision of suitable accommodation for older prisoners, health and social care on the prison estate, and upon the release of older prisoners, continuity of medical treatment or care in the community (HoC 2020). Further research to explore implementation of these measures would be welcomed to support a future strategy. Specific to older prisoners with dementia and mild cognitive impairment, a step-by-step care pathway and training materials were developed for use in UK prisons (Forsyth et al. 2020). Further research is required to explore the use of such a pathway in prisons including guidance required on specific screening tools, monitoring processes for those with dementia or cognitive impairment, prison environment and training resources. This would also support accurate identification of dementia or cognitive impairment prevalence within prisons.

## CPD reflective questions

1. What environmental adaptations could be made to support prisoners with dementia or cognitive impairment?
2. What factors would you need to consider when designing a dementia care pathway including staff training within a prison environment?
3. How would the above be evaluated?

## Figures and Tables

**Table 1:** Summary of themes and main findings

Theme	Summary of main findings
Prevalence of dementia in prisons	◾ Ranged from 0.8% - 18.8%. ◾ Different prison populations reported across studies. ◾ Diversity of screening and diagnostic tools implemented.
Identification of older prisoners needs	A need to support older prisoners with functional requirements and environment A suitable framework for staff to manage care for older prisoners with dementia. Suitable cognitive screening tools and referral practices in a transparent and consistent manner.
Knowledge of correctional officers and legal professionals	Prisoners identified as having a disability by officers tended to be older, have been hospitalised within the last 2 years and have a hearing impairment. Legal professionals have a lack of knowledge on cognitive impairment and training. Use of a standardised checklist may facilitate identification of cognitive impairment and disabilities.

## Key Points

◾ Prevalence of dementia in prisons ranged from 1 to 19%. The large variance may have been due to differences in study populations, screening tools and interpretation of scoring.
◾ Identifying the most appropriate screening tool for dementia in the prison population will support appropriate referrals, timely diagnosis and treatment.
◾ Dementia friendly environment features are recommended to support prisoners with dementia or cognitive impairment such as lighting and noise minimisation.
◾ All prison personnel require education and training in dementia to support prisoners with referrals and care within prison. Further research of training packages and care pathways within prisons are needed.
